# Pasteurized *Akkermansia muciniphila* alleviates high-fat diet-induced bone loss via Nr4a1-dependent Treg differentiation

**DOI:** 10.3389/fimmu.2026.1833607

**Published:** 2026-05-22

**Authors:** Si Chen, Chenyang Zhang, Xue Liu, Yiying Zhu, Chenguang Niu, Wanqi Lv

**Affiliations:** 1 Department of Multidisciplinary Consultant Center, Shanghai Stomatological Hospital and School of Stomatology, Fudan University, Shanghai, China; 2 Shanghai Key Laboratory of Craniomaxillofacial Development and Diseases, Fudan University, Shanghai, China; 3 Department of Oral Implantology, Shanghai Stomatological Hospital and School of Stomatology, Fudan University, Shanghai, China; 4 Department of Endodontics, Shanghai Ninth People’s Hospital, Shanghai Jiao Tong University School of Medicine, College of Stomatology, Shanghai Jiao Tong University, National Clinical Research Center for Oral Diseases, National Center for Stomatology, Shanghai Key Laboratory of Stomatology, Shanghai, China

**Keywords:** *Akkermansia muciniphila*, bone metabolism, gut microbiota, Nr4a1, obesity, osteoclastogenesis, Treg differentiation

## Abstract

**Background:**

Obesity, a global epidemic, disrupts bone metabolism via gut microbiota dysbiosis, and probiotic/postbiotic supplementation emerges as a promising intervention. Akkermansia muciniphila (Akk), a next-generation probiotic, exerts metabolic benefits in obesity, yet its effects on bone homeostasis—especially in pasteurized form (pAkk)—and underlying mechanisms remain unclear.

**Methods:**

High-fat diet (HFD)-induced obese mice were used to establish bone loss models, with fecal microbiota transplantation to verify gut microbiota’s role. Mice were gavaged with live Akk, pAkk, or control for 4 weeks. Bone microarchitecture was assessed via micro-computed tomography (μCT), and bone formation/resorption were detected by histomorphometry, ELISA, and TRAP staining. Flow cytometry, immunofluorescence, and qRT-PCR analyzed regulatory T (Treg) cell differentiation. RNA sequencing identified key genes, and Nr4a1 knockout mice validated the mechanism. Cell coculture confirmed pAkk-induced Tregs’ inhibitory effect on osteoclastogenesis.

**Results:**

Obesity-related gut microbiota induced trabecular bone loss, with reduced intestinal Akk abundance. pAkk (but not live Akk) rescued HFD-induced bone loss, increased bone formation marker (P1NP), decreased resorption marker (β-CTX), and inhibited osteoclast differentiation. pAkk promoted CD4^+^CD25^+^Foxp3^+^ Treg differentiation in the intestine and spleen via CD103^+^ dendritic cells, and these Tregs suppressed osteoclastogenesis. Transcriptomic and functional validation showed Nr4a1 was upregulated by pAkk and essential for Treg differentiation; Nr4a1 knockout abrogated pAkk’s bone-protective effects.

**Conclusion:**

Pasteurized Akkermansia muciniphila alleviates HFD-induced bone loss in obese mice by promoting intestinal and systemic Treg differentiation to inhibit osteoclastogenesis, dependent on the nuclear hormone receptor Nr4a1. Our findings identify pAkk as a promising postbiotic for obesity-related bone loss and uncover a novel Nr4a1/Treg axis linking gut microbiota to bone homeostasis.

## Graphical Abstract

**Figure:**
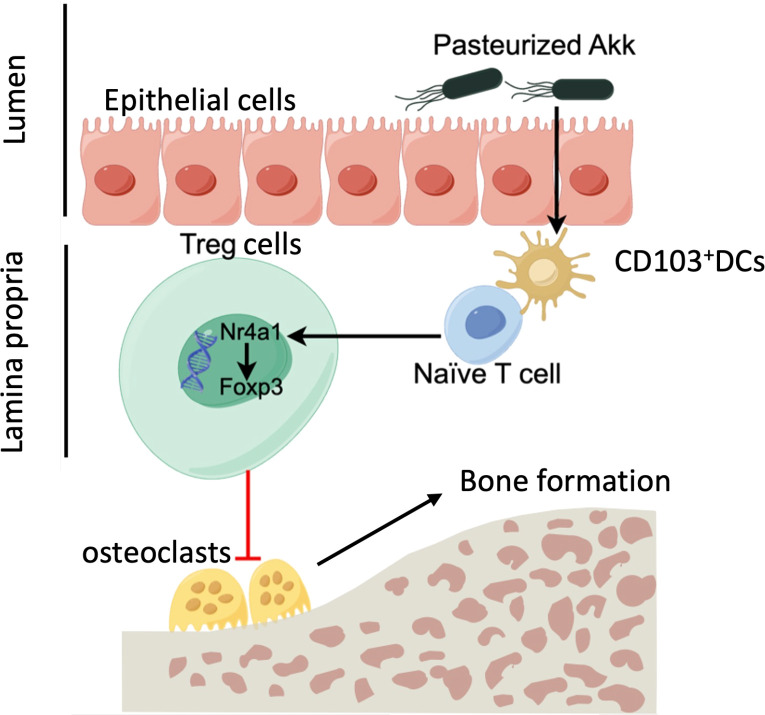

## 1 Introduction

Obesity is a global epidemic characterized by excessive adipose tissue accumulation, with projections indicating that over 1 billion individuals will be affected by 2030 (1). As a major public health concern, obesity is associated with a spectrum of comorbidities, including metabolic syndrome, cardiovascular diseases, and increased risk of bone fractures (2, 3). However, the underlying mechanisms by which obesity dysregulates bone metabolism remain incompletely elucidated. The gut microbiota, a complex microbial community residing in the gastrointestinal tract and referred to as the “second human genome”, has been established as a key regulator of bone remodeling and skeletal disorders such as osteoporosis (4, 5). Gut microbiota dysbiosis is a hallmark of obesity and overweight (6, 7), making microbiota-targeted strategies (e.g., probiotic supplementation) a promising avenue for treating obesity-related skeletal diseases.

*Akkermansia muciniphila* (Akk), a mucin-degrading commensal bacterium, is a novel next-generation probiotic with well-documented metabolic benefits (8, 9). Supplementation with Akk or its pasteurized form improves insulin sensitivity and reduces hyperinsulinemia in obese human volunteers, with pAkk exhibiting superior efficacy compared to viable Akk (10). Additionally, Akk or its pilus-like protein Amuc_1100 exerts a protective effect against alveolar bone loss induced by periodontal pathogens (11). However, whether Akk can regulate bone remodeling in obesity, and the effective form (live vs. pasteurized) and underlying mechanisms of its action, remain unknown (12). The gut microbiota is the largest source of intrinsic non-self-antigens continuously sensed by the host immune system; as a key commensal microbe (constituting 1%–4% of the human intestinal microbiota), Akk maintains metabolic homeostasis in part through immune regulation (13). HFD-induced obese mice exhibit increased adipose tissue-resident Tregs following Akk administration, accompanied by improved glucose tolerance (14), and Akk-derived antigens can reprogram naive CD4^+^ T cells in the intestinal mucosa to differentiate into Tregs (15–17).

Bone remodeling is a tightly coordinated process mediated by two major cell types: osteoblasts (responsible for bone formation) and osteoclasts (mediating bone resorption) (18). The interaction between T lymphocytes and osteoclast lineage cells is a central focus of osteoimmunology, a discipline that integrates bone biology and immunology (19, 20). CD4^+^CD25^+^Foxp3^+^ Tregs, a subset of CD4^+^ T cells with immunosuppressive functions, are critical for maintaining bone homeostasis primarily by inhibiting osteoclast differentiation—they do so by suppressing the production of receptor activator of nuclear factor-κB ligand (RANKL) and macrophage colony-stimulating factor (M-CSF), thereby increasing bone mass (21–23).

In this study, we aimed to investigate the effects and molecular mechanisms of Akk on bone remodeling in HFD-induced obesity. We found that obesity-related gut microbiota (with reduced Akk abundance) accelerates bone loss in mice. Supplementation with pAkk, but not live Akk, significantly prevented HFD-induced bone loss by promoting Treg differentiation and subsequent osteoclast inhibition. Furthermore, the nuclear hormone receptor Nr4a1 was essential for pAkk-mediated Treg differentiation and osteoclast suppression, and Nr4a1 knockout abrogated the bone-protective effects of pAkk in obese mice. Our findings reveal a novel gut microbiota-immune-bone axis and identify pAkk as a promising postbiotic for the treatment of obesity-related bone loss.

## 2 Materials and methods

### 2.1 Animal experiments

All animal experiments were approved by the Committee for the Care and Use of Laboratory Animals at Fudan University (Approval No.: 202202006S, Shanghai, China) and strictly conformed to the ARRIVE (Animal Research: Reporting *In Vivo* Experiments) guidelines. Mice were group-housed in a specific pathogen-free (SPF) facility under a controlled environment (22 ± 2 °C, 50 ± 10% humidity) with a strict 12 h light/dark cycle and ad libitum access to food and water. A minimum of six mice per group were used based on our prior experience and common practice in comparable mouse bone studies, with the aim of providing adequate sensitivity while minimizing animal use in accordance with ARRIVE principles. No formal *a priori* power analysis was performed.

For the obesity model, 8-week-old male C57BL/6 mice were fed a HFD (60% kcal from fat, D12492, Research Diets) for 12 weeks; age-matched male mice fed a normal diet (ND, 10% kcal from fat, D12450J, Research Diets) served as controls.

### 2.2 Gut microbiota transplantation

8-week-old male C57BL/6 mice fed ND were used as recipients. Recipient mice were treated with a cocktail of antibiotics (ampicillin 1 g/L, vancomycin 0.5 g/L, neomycin 1 g/L, metronidazole 1 g/L) in drinking water for 2 weeks to deplete the endogenous intestinal microbiota (24). Donor mice were 8-week-old male C57BL/6 mice fed ND or HFD for 12 weeks. Fresh fecal pellets from donors were collected every 2 days, immediately homogenized in anaerobic phosphate-buffered saline (PBS), and centrifuged at 300 × g for 5 min to remove large debris. The supernatant (fecal microbiota suspension) was immediately administered to recipient mice by oral gavage (200 μL per mouse) every 2 days for 4 weeks. Recipient groups were designated as GMT^ND^ (microbiota from ND-fed donors) and GMT^HFD^ (microbiota from HFD-fed donors).

### 2.3 Akk or pAkk administration

8-week-old male C57BL/6 mice were fed HFD for 12 weeks and then randomly divided into three groups (n=6–8 per group) with no significant difference in body weight: Akk group (daily gavage of 2×10^8^ CFU Akk in 150 μL PBS containing 2.5% glycerol), pAkk group (daily gavage of 2×10^8^ CFU pAkk in 150 μL PBS containing 2.5% glycerol), and control group (daily gavage of 150 μL PBS containing 2.5% glycerol). Gavage was continued for 4 weeks, and mice were euthanized for sample collection at the end of treatment.

### 2.4 Identification of Nr4a1 knockout mice

Nr4a1 knockout (Nr4a1^-^/^-^) mice on a C57BL/6 background were purchased from Cyagen Biosciences (Shanghai, China); wild-type (WT) littermates were used as controls. Genomic DNA was extracted from mouse tail tissue using the TaKaRa MiniBEST Universal Genomic DNA Extraction Kit (TaKaRa, Shiga, Japan). Genotyping was performed by polymerase chain reaction (PCR) with the following specific primers: Forward: 5’-CAGTGGTGCTCAGAAACAGAAAAC-3’; Reverse: 5’-CAGACTAAGGACGAGCTGGGTG-3’. PCR products were separated by 2% agarose gel electrophoresis and visualized under ultraviolet light. For pAkk administration experiments, 8-week-old male Nr4a1^-^/^-^ and WT mice were fed HFD for 12 weeks, then gavaged daily with pAkk (2×10^8^ CFU/150 μL) or PBS control for 4 weeks.

### 2.5 Akk culture and pasteurization

*Akkermansia muciniphila* (ATCC BAA-835) was kindly provided by Prof. Yi Zhang (Peking University, Beijing, China). Akk was cultured anaerobically (80% N_2_, 20% CO_2_) at 37 °C in brain heart infusion (BHI) broth supplemented with 0.05% L-cysteine-HCl and 0.5% porcine mucin (both from Sigma-Aldrich, St. Louis, MO, USA). Bacteria were cultured to the late exponential growth phase (48 h), and bacterial concentration was determined by measuring absorbance at 600 nm (OD_600_). Pasteurized Akk (pAkk) was prepared by heating the bacterial suspension at 70 °C for 30 min, followed by cooling to room temperature; pAkk was stored at 4 °C and used within 1 week.

### 2.6 16S rRNA gene sequencing and bioinformatics analysis

Fecal samples were collected from ND- and HFD-fed mice, and total microbial genomic DNA was extracted using the PF Mag-Bind Stool DNA Kit (Omega Bio-tek, Norcross, GA, USA). The hypervariable V3-V4 region of the bacterial 16S rRNA gene was amplified with the universal primers 338F (5’-ACTCCTACGGGAGGCAGCAG-3’) and 806R (5’-GGACTACHVGGGTWTCTAAT-3’) on an ABI GeneAmp^®^ 9700 PCR thermocycler (ABI, CA, USA). Purified PCR amplicons were pooled in equimolar amounts and subjected to paired-end sequencing on the Illumina PE250 platform (Illumina, San Diego, CA, USA) by Majorbio Bio-Pharm Technology Co., Ltd. (Shanghai, China). Raw FASTQ files were demultiplexed, quality-filtered (fastp v0.19.6), and merged (FLASH v1.2.11). Operational taxonomic units (OTUs) were clustered at a 97% sequence similarity level using UPARSE v11. Taxonomic annotation was performed against the SILVA database, and bioinformatic analysis (alpha/beta diversity, microbial composition) was conducted on the Majorbio Cloud Platform (https://cloud.majorbio.com).

### 2.7 MicroCT measurements

After euthanasia of mice, femurs were dissected and fixed in 4% paraformaldehyde for 24 h. Ex vivo μCT analysis was performed on femurs using a Scanco μCT-40 scanner (Scanco Medical, Zurich, Switzerland) with the following parameters: 70 kVp X-ray tube voltage, 114 μA current, 200 ms integration time, and 12 μm voxel size. For trabecular bone analysis, a region of interest (ROI) consisting of 100 consecutive slices was selected starting 50 slices below the distal femoral growth plate. For cortical bone analysis, an ROI of 80 consecutive slices was selected at the femoral midshaft. Bone microarchitectural parameters were quantified using Scanco analysis software, including trabecular bone volume fraction (BV/TV), trabecular number (Tb.N), trabecular thickness (Tb.Th), trabecular separation (Tb.Sp), structure model index (SMI), and cortical thickness (Ct.Th).

### 2.8 Histomorphometric analysis

Femurs were fixed in 4% paraformaldehyde for 24 h, then decalcified in 10% ethylenediaminetetraacetic acid (EDTA) (pH 7.4) for 4 weeks (with EDTA changed every 3 days). Decalcified samples were dehydrated through a graded ethanol series, cleared in xylene, embedded in paraffin, and sectioned into 5 μm serial longitudinal sections. Hematoxylin and eosin (H&E) staining was performed to evaluate trabecular bone microstructure. Tartrate-resistant acid phosphatase (TRAP) staining was conducted using a commercial kit (Servicebio, Wuhan, China) according to the manufacturer’s instructions to identify multinucleated osteoclasts (TRAP^+^ cells with ≥3 nuclei).

### 2.9 Quantitative bone histomorphometry

Bone histomorphometric measurements were performed in accordance with the guidelines of the Nomenclature Committee of the American Society of Bone and Mineral Research (ASBMR) (25). Mice were injected subcutaneously with calcein (25 μg/g body weight, Sigma-Aldrich) on day 10 and day 3 before euthanasia for dynamic bone histomorphometry. Femurs were fixed in periodate-lysine-paraformaldehyde (PLP) fixative for 24 h, dehydrated in absolute ethanol for 3 days, incubated in acetone at -20 °C overnight, and embedded in methyl methacrylate (MMA). 8 μm sagittal sections were cut using a Leica CM 1950 cryostat microtome (Leica, Wetzlar, Germany) and mounted for fluorescence microscopy. Static and dynamic bone parameters were quantified using the Bioquant Image Analysis System (R&M Biometrics, Nashville, TN, USA), including mineralizing surface/bone surface (MS/BS) and mineral apposition rate (MAR).

### 2.10 Enzyme-linked immunosorbent assay

Blood samples were collected from the orbital sinus of mice, and serum was separated by centrifugation at 3000 × g for 15 min at 4 °C. Serum levels of procollagen I N-terminal propeptide (P1NP, a bone formation marker) and β-isomer of C-terminal telopeptide of type I collagen (β-CTX, a bone resorption marker) were measured using commercial ELISA kits (Elabsience, Wuhan, China) according to the manufacturer’s instructions. All samples were assayed in duplicate, and standard curves were generated for quantitative analysis.

### 2.11 Immunofluorescence assay

Colon tissues were collected, embedded in optimal cutting temperature (OCT) compound, and frozen in liquid nitrogen. 5 μm frozen sections were cut, fixed in cold acetone for 10 min, and blocked with 5% bovine serum albumin (BSA) in PBS for 1 h at room temperature. Sections were incubated with primary antibodies overnight at 4 °C: anti-Foxp3 (eFluor™570-conjugated, #41-4777-82, Invitrogen, CA, USA), anti-CD103 (#ab224202, Abcam, Cambridge, MA, USA), and anti-Nr4a1 (#3960, Cell Signaling Technology (CST), MA, USA). For CD103 and Nr4a1 staining, sections were further incubated with Alexa Fluor^®^488-conjugated anti-rabbit secondary antibody (#4412, CST) for 1 h at room temperature. Nuclei were counterstained with 4’,6-diamidino-2-phenylindole (DAPI). Images were acquired using a Leica TCS SP8 confocal fluorescence microscope (Leica, Germany), and positive cell counts were quantified using ImageJ software (NIH, Bethesda, MD, USA) (five random fields per section, three sections per sample).

### 2.12 Flow cytometry

Colonic lamina propria (cLP) and spleen tissues were collected, and single-cell suspensions were prepared by mechanical dissociation and passage through a 40 μm nylon mesh filter. Red blood cells (RBCs) were lysed with RBC lysis buffer (eBioscience, San Diego, CA, USA) for 15 min at 4 °C in the dark. For Treg analysis, cells were stained with FITC-conjugated anti-CD4 and APC-conjugated anti-CD25 (both from eBioscience) for 30 min at 4 °C in the dark, then fixed and permeabilized using the Foxp3 Staining Buffer Set (eBioscience) followed by intracellular staining with PE-conjugated anti-Foxp3 (eBioscience). For Th17 cell analysis, cells were stimulated with a cell activation cocktail (containing brefeldin A, eBioscience) for 5 h at 37 °C, then stained with PE-anti-CD45 and FITC-anti-CD4 (both from eBioscience), fixed/permeabilized, and stained intracellularly with PE-Cy7-anti-IL-17A (eBioscience). Flow cytometry data were acquired on a BD FACS Aria II flow cytometer (BD Biosciences, San Jose, CA, USA) and analyzed using FlowJo v7.6.1 software (BD Biosciences).

### 2.13 Treg isolation and naïve T-cell isolation

CD4^+^CD25^+^Foxp3^+^ Tregs were sorted from the cLP of mice by fluorescence-activated cell sorting (FACS) using a BD FACS Aria II flow cytometer (purity >95%). Naïve CD4^+^ T cells (CD4^+^CD62L^+^CD25^-^CD44^-^) were isolated from the spleens of 8-week-old male C57BL/6 mice by FACS (purity >98%). Isolated naïve T cells were seeded in 24-well plates and stimulated with live Akk or pAkk at a multiplicity of infection (MOI) of 100 for 2 h (Akk) or 24 h (pAkk) at 37 °C in a 5% CO_2_ incubator.

### 2.14 Cell coculture and osteoclast differentiation

Bone marrow cells were flushed from the femurs and tibias of 8-week-old male C57BL/6 mice with PBS, and single-cell suspensions were prepared. Cells were cultured in α-minimal essential medium (α-MEM) supplemented with 10% fetal bovine serum (FBS), 0.1% penicillin-streptomycin, and 10 ng/mL M-CSF (PeproTech, Rocky Hill, NJ, USA) for 24 h at 37 °C in a 5% CO_2_ incubator. Non-adherent cells (BMMs) were collected, seeded in 96-well plates at a density of 5×10^4^ cells/well, and cocultured with sorted CD4^+^CD25^+^Foxp3^+^Tregs (1×10^4^ cells/mL) in the presence of 10 ng/mL M-CSF and 50 ng/mL RANKL (PeproTech) for 5 days (medium changed every 48 h). Osteoclast differentiation was assessed by TRAP staining (Sigma-Aldrich), and TRAP^+^ multinucleated cells (≥3 nuclei) were counted as osteoclasts (five random fields per well, three replicate wells per sample).

### 2.15 RNA extraction and quantitative real-time PCR

Total RNA was extracted from stimulated naïve T cells and sorted Tregs using TRIzol^®^ reagent (Invitrogen, Carlsbad, CA, USA) according to the manufacturer’s instructions. cDNA was synthesized from 1 μg of total RNA using the SuperScript First-Strand cDNA Synthesis Kit (Invitrogen). qRT-PCR was performed using SYBR Green Master Mix (Roche Applied Science, Mannheim, Germany) on a 7500 Real-Time PCR System (Thermo Scientific, Waltham, MA, USA). The thermal cycling conditions were: 95 °C for 10 min (pre-denaturation), followed by 40 cycles of 95 °C for 15 s (denaturation) and 65 °C for 1 min (annealing/extension). Gapdh was used as the housekeeping gene for normalization, and relative gene expression was calculated using the 2^-^ΔΔCt method. Primer sequences are listed in **Supplementary Table 1**.

### 2.16 RNA sequencing and bioinformatics analysis

Colon tissues were collected from control, Akk, and pAkk groups, and total RNA was extracted using TRIzol^®^ reagent (Invitrogen). Genomic DNA was removed using DNase I (TaKaRa), and RNA quality was assessed using a NanoDrop 2000 spectrophotometer (Thermo Scientific) and Agilent 2100 Bioanalyzer (Agilent Technologies, Santa Clara, CA, USA). RNA-seq libraries were constructed using the TruSeq™ RNA Sample Preparation Kit (Illumina) with 1 μg of high-quality total RNA and sequenced on the Illumina NovaSeq 6000 platform (Illumina) by Majorbio Bio-Pharm Technology Co., Ltd. Raw paired-end reads were quality-filtered with fastp, and clean reads were aligned to the mouse reference genome (mm10) using HISAT2. Transcript assembly and quantification were performed with StringTie and RSEM, respectively. Differentially expressed genes (DEGs) were identified with the criteria of |log_2_(fold change)| ≥1 and adjusted P-value (P-adjust) ≤0.05. Gene Ontology (GO) and Kyoto Encyclopedia of Genes and Genomes (KEGG) enrichment analyses of DEGs were performed using the clusterProfiler R package, with statistical significance defined as P-adjust ≤0.05. Given the exploratory nature of the RNA-seq analysis and the modest sample size (n = 4 per group), transcriptomic findings were used primarily for candidate screening and were further validated by independent approaches, including qRT-PCR, immunofluorescence, functional Treg assays, and Nr4a1 knockout experiments.

### 2.17 Statistical analysis

All statistical analyses were performed using GraphPad Prism 9.0 software (GraphPad Software, San Diego, CA, USA). Data are presented as the mean ± standard error of the mean (SEM). Differences between two groups were analyzed using the unpaired Student’s t-test. Differences among three or more groups were analyzed using one-way or two-way analysis of variance (ANOVA) followed by the Bonferroni *post hoc* test for multiple comparisons. A P-value <0.05 was considered statistically significant. The number of animals/cell replicates and specific statistical tests used are indicated in the Figure legends.

## 3 Results

### 3.1 Obesity-related gut microbiota induces bone impairment and reduces intestinal Akk abundance

**Figure 1:**
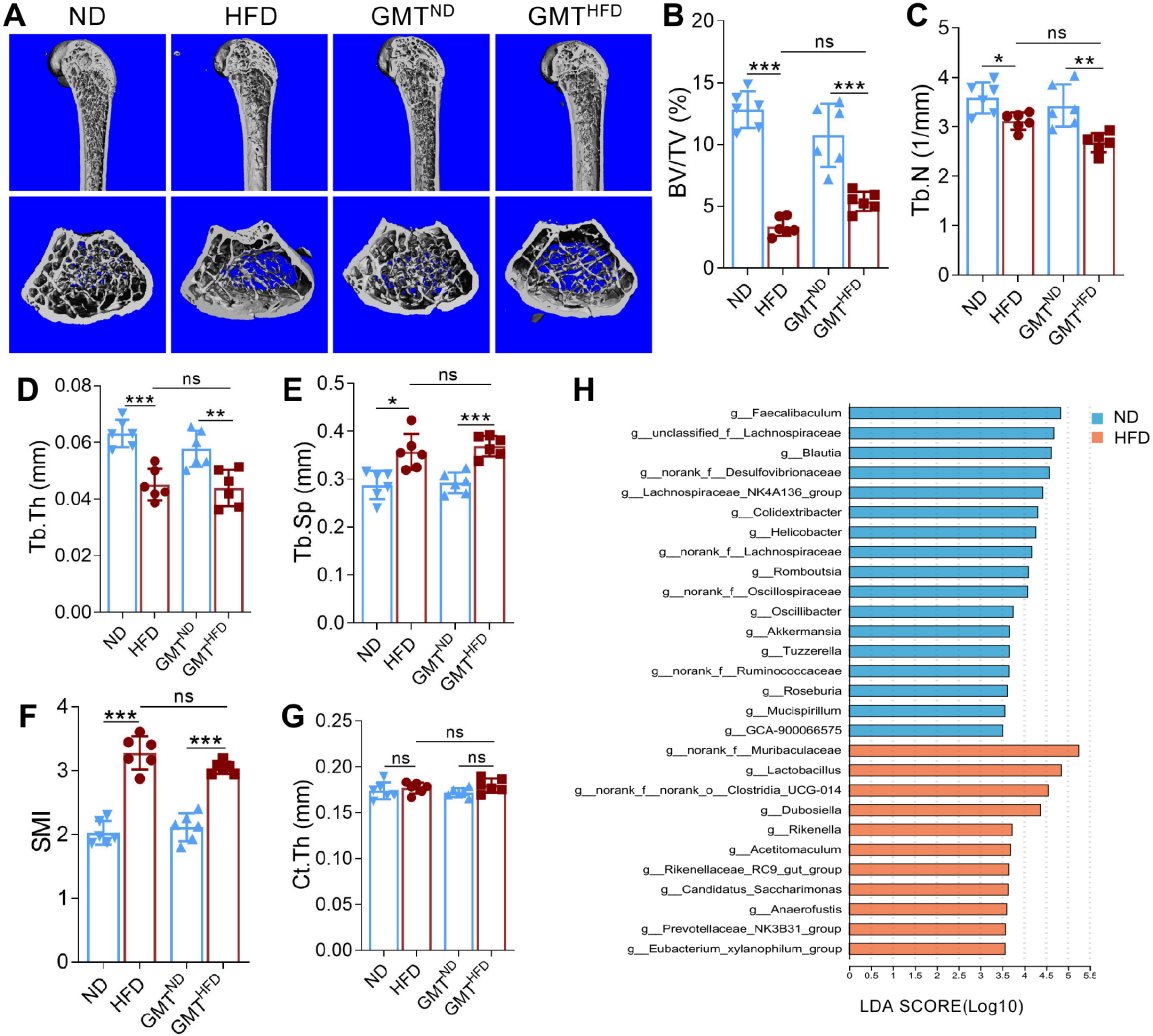
Gut microbiota transplanted from HFD mice accelerated bone loss with Akk decreased. **(A)** Representative images of femoral bone structures assessed by μCT in mice fed with normal diet (ND), high-fat diet (HFD), and gut microbiota transplantation from ND(GMT^ND^) or HFD (GMT^HFD^). **(B–G)** μCT measurements of trabecular bone volume fraction (BV/TV), trabecular number (Tb.N), trabecular thickness (Tb.Th), trabecular separation (Tb.Sp), structure model index (SMI), and cortical thickness (Ct.Th). **(H)** Linear discriminant analysis (LDA) score plot of differentially abundant genus between the ND and HFD groups (LDA score for discriminative features > 3.5). Each dot indicates an individual mouse (n = 6 per group). Data are shown as means ± SEM and analyzed by one-way ANOVA followed by Bonferroni *post hoc* test. ns, not significant; *P < 0.05; **P < 0.01, and ***P < 0.001.

To investigate the role of gut microbiota in obesity-associated bone metabolism, we performed fecal microbiota transplantation (FMT) from ND- or HFD-fed donor mice to antibiotic-depleted ND recipient mice (GMT^ND^ and GMT^HFD^ groups).μCT analysis of femoral bones revealed significant trabecular bone loss in both HFD-fed and GMT^HFD^ recipient mice compared to ND and GMT^ND^ mice (**Figure 1A**), characterized by reduced BV/TV, Tb.N, and Tb.Th (**Figures 1B–D**); no significant difference in bone parameters was observed between the HFD and GMT^HFD^ groups. Additionally, GMT^HFD^ mice exhibited significantly increased Tb.Sp and SMI (markers of bone deterioration) compared to GMT_ND mice, while Ct.Th (cortical bone parameter) was unaltered among all groups (**Figures 1E–G**). 16S rRNA gene sequencing of fecal samples revealed distinct gut microbial communities between ND and HFD mice (**Supplementary Figures 1A–C**), with a significant reduction in the relative abundance of Akk in HFD-fed mice (**Figure 1H**) These results indicate that obesity-related gut microbiota drives bone loss in mice, and reduced Akk abundance is a feature of the obese gut microbiome.

### 3.2 Pasteurized Akk rescues HFD-induced bone loss in obese mice

**Figure 2:**
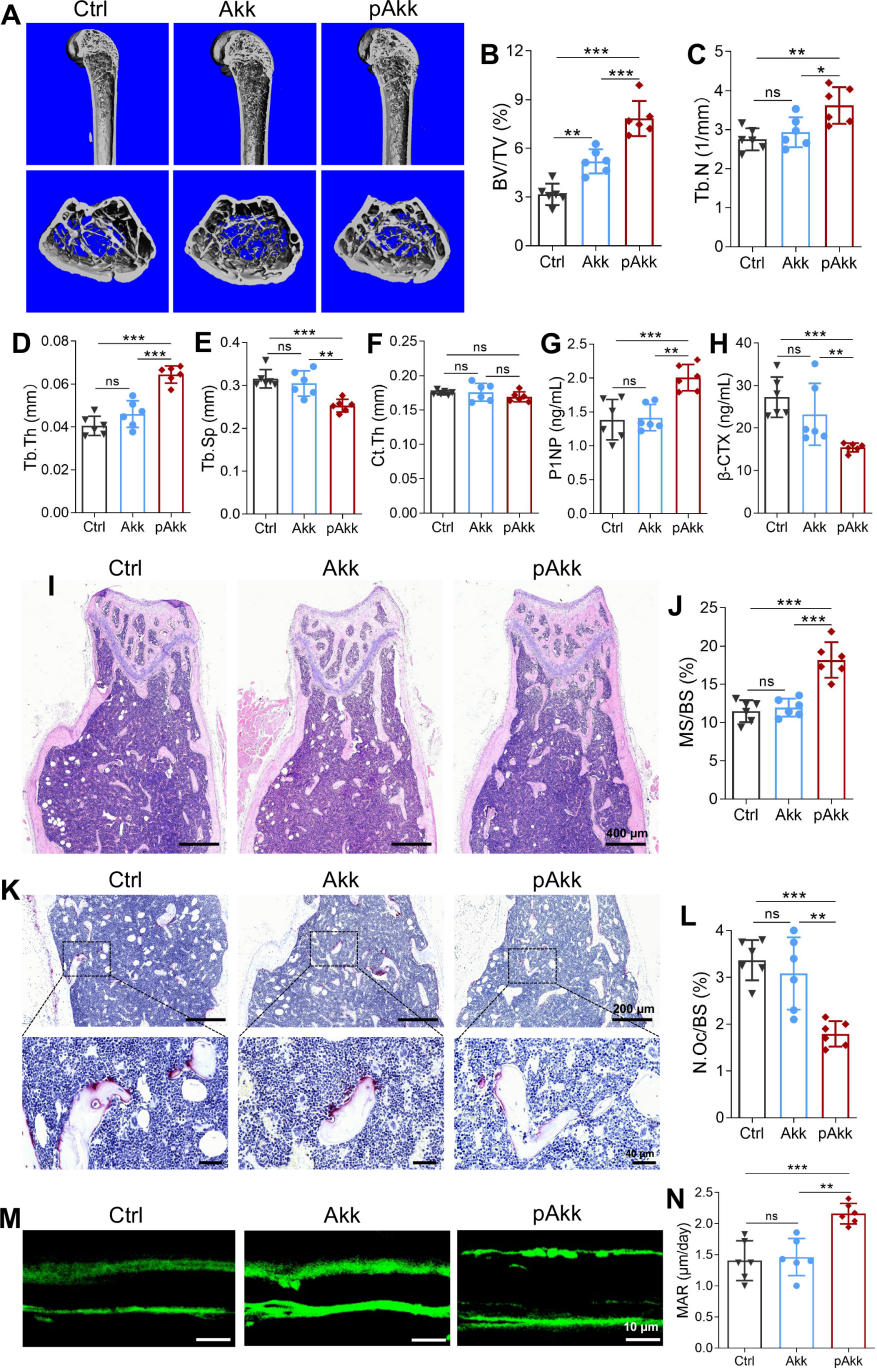
Supplementation with pAkk prevented bone loss in HFD mice. **(A)** Representative images of femoral bone structures assessed by μCT in HFD mice administered with Akk, pAkk, and the control. **(B-F)** μCT measurements of trabecular bone volume fraction (BV/TV), trabecular number (Tb.N), trabecular thickness (Tb.Th), trabecular separation (Tb.Sp), and cortical thickness (Ct.Th). **(G, H)** Serum levels of N-terminal propeptide of type I procollagen (P1NP), and β-terminal telopeptide (β-CTX) measured by ELISA. **(I)** Representative images of hematoxylin and eosin (H&E) staining of the distal femur bone in HFD mice administered with Akk, pAkk, and the vehicle control (Scale bar: 400 μm). **(J)** Histomorphometric indices of mineralizing surface/bone surface (MS/BS). **(K)** Representative images of tartrate resistant acid phosphatase (TRAP)-stained sections of the distal femur bone, and a higher magnification was shown in lower panel (Scale bar: upper panel 100 μm, lower panel 40 μm). **(L)** Histomorphometric indices of osteoclasts per bone surface (N.Oc/BS). **(M)** Trabecular calcein double-fluorescence labeling images (green) of the representative sections (Scale bar: 10 μm). **(N)** Quantification of mineral apposition rate (MAR). Each dot indicates an individual mouse (n = 6 per group). Data are shown as means ± SEM and analyzed by one-way ANOVA followed by Bonferroni *post hoc* test. ns, not significant; *P < 0.05; **P < 0.01, and ***P < 0.001.

Given the key role of gut microbiota in obesity-related bone remodeling and the reduced Akk abundance in obese mice, we investigated the therapeutic effects of live and pasteurized Akk on bone metabolism in HFD-fed mice. μCT analysis demonstrated that pAkk administration significantly improved femoral trabecular bone microarchitecture in HFD-fed mice (**Figure 2A**), with increased BV/TV, Tb.N, and Tb.Th, and decreased Tb.Sp compared to the control and live Akk groups (**Figures 2B–E**). Live Akk administration only slightly increased BV/TV with no significant effects on other trabecular bone parameters, and Ct.Th was unaltered among the three groups (**Figure 2F**). Serum ELISA assays showed that pAkk administration significantly increased P1NP (bone formation) levels and decreased β-CTX (bone resorption) levels in HFD-fed mice, while live Akk had no such effects (**Figures 2G, H**).

Histological analysis confirmed the bone-protective effects of pAkk: H&E staining revealed more intact trabecular bone structures in pAkk-treated mice (**Figure 2I**), with a 158.45% increase in MS/BS compared to the control group (**Figure 2J**). TRAP staining showed a marked reduction in the number of multinucleated osteoclasts in the distal femur of pAkk-treated mice compared to the control and live Akk groups (**Figure 2K**), with significantly decreased N.Oc/BS (**Figure 2L**). Dynamic bone histomorphometry (calcein double-labeling) demonstrated a significantly elevated MAR (a marker of bone formation rate) in pAkk-treated mice, while no significant difference in MAR was observed between the live Akk and control groups (**Figures 2M, N**). Collectively, these results indicate that pAkk, but not live Akk, exerts a protective effect against HFD-induced trabecular bone loss by inhibiting osteoclastogenesis and promoting bone formation in obese mice.

### 3.3 pAkk promotes Treg differentiation in the intestine and periphery to inhibit osteoclastogenesis

**Figure 3:**
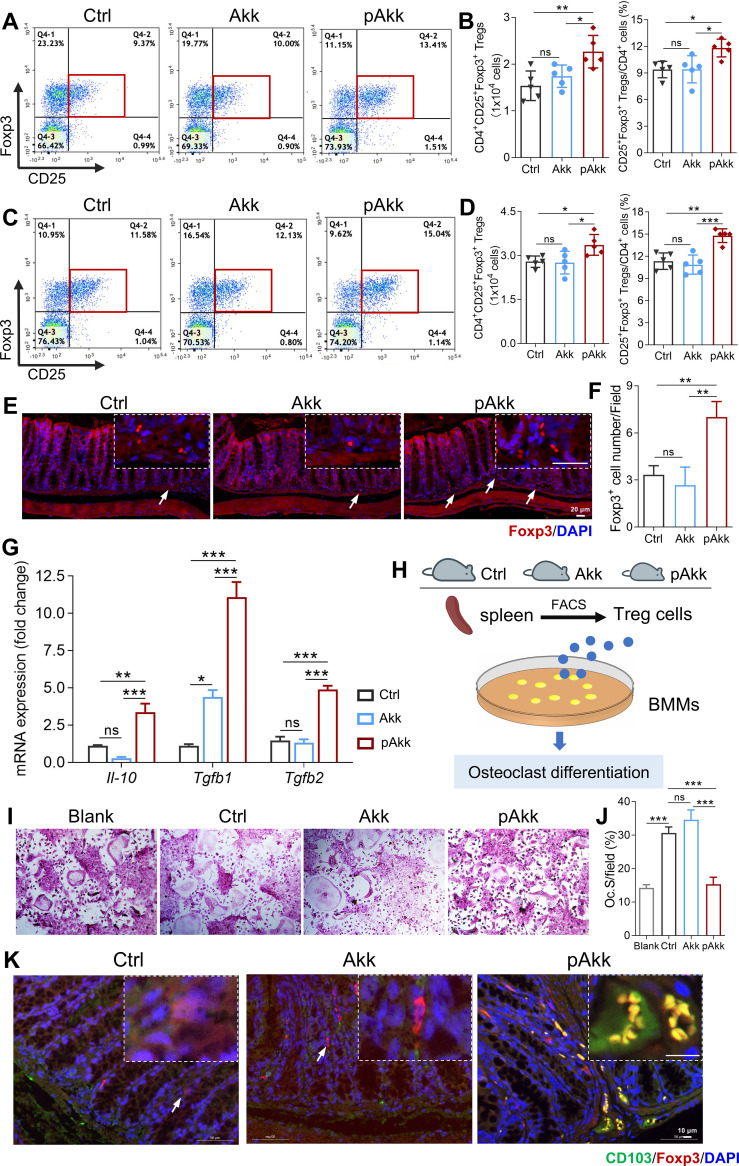
Supplementation with pAkk increased CD4^+^CD25^+^Foxp3^+^ Tregs in the intestine to inhibit osteoclastogenesis. **(A, B)** Representative plots and quantification of CD25^+^Foxp3^+^Tregs in CD4^+^T cells of colonic lamina propria (cLP) from HFD mice administered with Akk, pAkk, and the control assessed using flow cytometry. **(C, D)** Representative plots and quantification of CD25^+^Foxp3^+^Tregs in CD4^+^T cells of spleen from HFD mice administered with Akk, pAkk, and the control assessed using flow cytometry. **(E)** Immunostaining against Foxp3 in the colon tissues, and the white arrow indicated positive cells (Scale bar: 20 μm). **(F)** Quantification of Foxp3^+^cells in the colon tissues. **(G-J)** Foxp3^+^Tregs were purified from spleen of HFD mice administered with Akk, pAkk, and the control by fluorescence-activated cell sorting (FACS). **(G)** Relative expression of *Il-10*, *Tgfb1* and *Tgfb2* at mRNA level in Foxp3^+^Tregs analyzed using qRT-PCR. **(H)** Foxp3^+^Tregs were co-cultured with bone marrow monocytes (BMMs) for osteoclast induction. **(I)** Representative images of TRAP staining. **(J)** Quantification of osteoclast surface per bone surface (OcS/BS). **(K)** Immunofluorescence analysis of CD103^+^DCs and Foxp3^+^Tregs in mice colonic tissues of the control, Akk and pAkk groups (Scale bar: 10 μm). Blue, nucleus; green, CD103; red, Foxp3. Each dot indicates an individual mouse (n = 5 per group). Data are shown as means ± SEM and analyzed by one-way ANOVA followed by Bonferroni *post hoc* test. ns, not significant; *P < 0.05; **P < 0.01, and ***P < 0.001.

Akk modulates systemic homeostasis through immune regulation, and immune-bone cross-talk is critical for bone remodeling (26). We therefore investigated the effects of pAkk on intestinal and systemic immune responses in HFD-fed mice (Treg/Th17 gating strategy in **Supplementary Figure 2A**). Flow cytometry analysis revealed a significant increase in the frequency and absolute number of CD4^+^CD25^+^Foxp3^+^ Tregs in both the cLP and spleen of pAkk-treated HFD mice compared to the control and live Akk groups (**Figures 3A–D**), while the frequency of CD4^+^IL-17A^+^ Th17 cells was unaltered in the cLP and spleen among all groups (**Supplementary Figures 2B–E**). Immunofluorescence staining of colon tissues confirmed increased Foxp3^+^ Treg infiltration in pAkk-treated mice (**Figures 3E, F**). qRT-PCR analysis of sorted cLP Tregs showed that pAkk administration significantly upregulated the expression of Treg-associated anti-inflammatory cytokines (*Il-10*, *Tgfb1*, *Tgfb2*)—which are critical for osteoclast inhibition—while live Akk had no significant effect (**Figure 3G**).

To confirm that pAkk-induced Tregs directly inhibit osteoclastogenesis, we sorted Tregs from the spleens of control, Akk, and pAkk-treated mice and cocultured them with BMMs in the presence of M-CSF and RANKL. TRAP staining showed that Tregs from pAkk-treated mice significantly inhibited BMM differentiation into osteoclasts, while Tregs from live Akk-treated mice had no such suppressive effect (**Figures 3H–J**). CD103^+^ DCs are key intestinal antigen-presenting cells that induce Treg differentiation and immune tolerance to commensal microbiota (27). Immunofluorescence co-staining of colon tissues revealed a significant increase in CD103^+^DC-Foxp3^+^Treg colocalization in pAkk-treated mice compared to the control and live Akk groups (**Figure 3K**). These results indicate that pAkk likely engages intestinal CD103^+^ DCs to promote Treg differentiation in the intestine and periphery, and these induced Tregs subsequently inhibit osteoclast differentiation.

### 3.4 pAkk enhances intestinal Treg differentiation via upregulation of the nuclear hormone receptor Nr4a1

**Figure 4:**
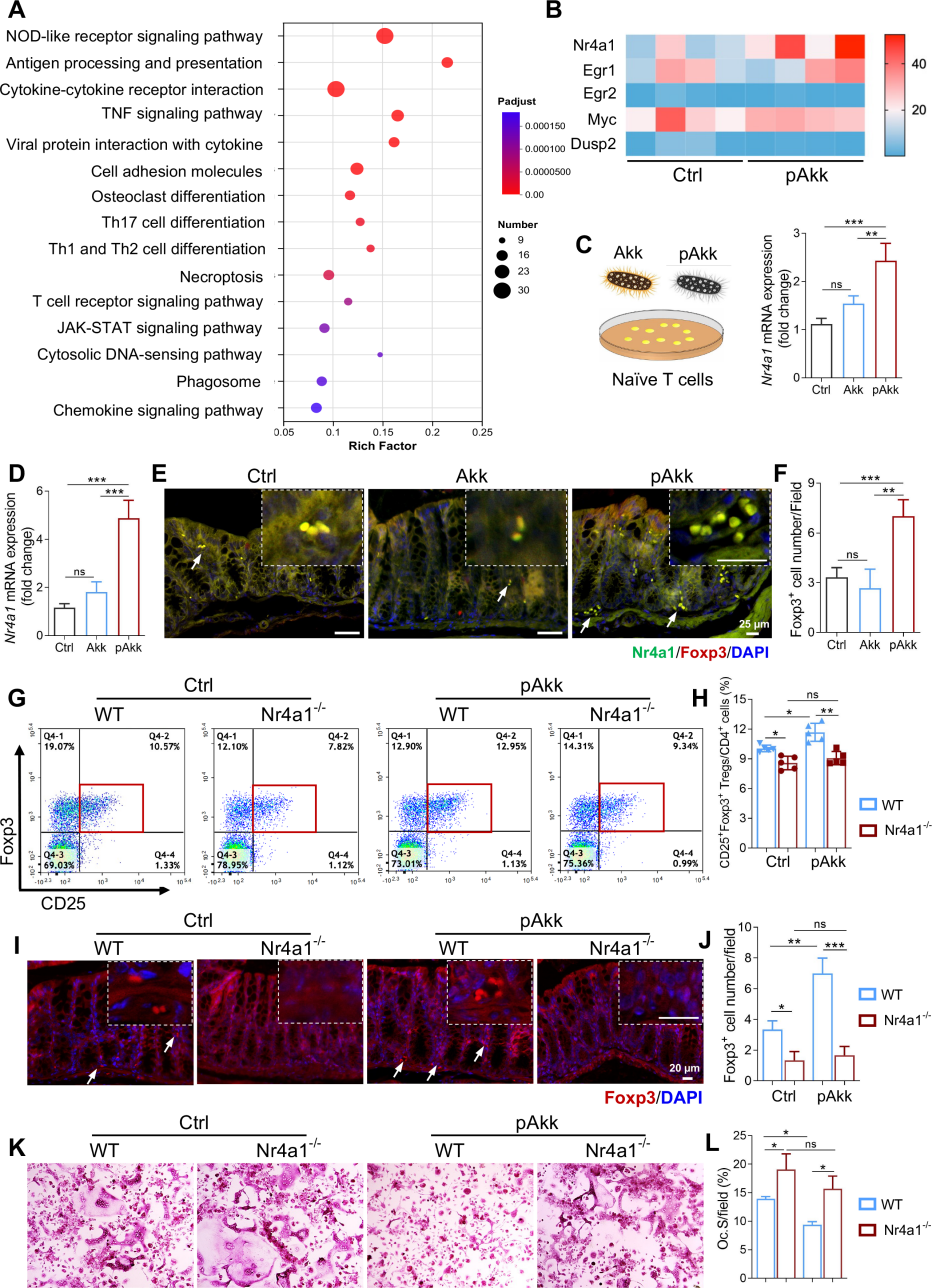
pAkk enhanced intestinal Treg differentiation by up-regulating Nr4a1. **(A, B)** The colon tissues collected from mice in control, Akk and pAkk groups were subjected to transcriptome sequencing (n = 4 per group). **(A)** The significantly enriched KEGG terms between pAkk and control groups. **(B)** Heatmap analysis with genes associated with early TCR responses. **(C)** Relative mRNA expression of *Nr4a1* in the naïve T cells measured with qRT-PCR. **(D)** Relative mRNA expression of *Nr4a1* in the colon tissues from HFD mice administered with Akk, pAkk, and the control. **(E)** Immunostaining against Nr4a1 and Foxp3 in the colon tissues, and the white arrow indicated positive cells (Scale bar: 25 μm). **(F)** Quantification of Nr4a1^+^/Foxp3^+^ co-stained cells in the colon tissues. **(G-L)** Nr4a1KO (Nr4a1^-/-^) and wild-type (WT) mice were fed with high-fat diet (HFD) for 12 weeks, and further administered with pAkk by daily oral gavage for 4 weeks. **(G)** Representative plots of CD25^+^Foxp3^+^Tregs in CD4^+^T cells assessed using flow cytometry. **(H)** Percentage of CD25^+^Foxp3^+^Tregs in CD4^+^T cells assessed using flow cytometry (n = 5 per group). **(I)** Representative immunostaining against Foxp3 in the colon tissues (Scale bar: 20 μm). **(J)** Quantification of Foxp3^+^cells in the colon tissues. **(K, L)** Foxp3^+^Tregs were purified from spleen of Nr4a1^-/-^ mice and WT littermates administered with pAkk or the control by fluorescence-activated cell sorting (FACS) and co-cultured with bone marrow monocytes (BMMs) for osteoclast induction. **(K)** Representative images of TRAP staining. **(L)** Quantification of osteoclast surface per bone surface (OcS/BS). Data are shown as means ± SEM and analyzed by two-way ANOVA followed by Bonferroni *post hoc* test. ns, not significant; *P < 0.05; **P < 0.01, and ***P < 0.001.

To identify the molecular mechanism underlying pAkk-induced Treg differentiation, we performed RNA-seq on colon tissues from control, Akk, and pAkk-treated mice. Transcriptomic analysis revealed distinct gene expression profiles among the three groups (**Supplementary Figure 3A**), with 70 upregulated and 505 downregulated DEGs in pAkk-treated mice compared to controls (**Supplementary Figures 3B, C**). KEGG enrichment analysis of DEGs showed significant enrichment of immune-related pathways, including antigen processing and presentation and the T cell receptor (TCR) signaling pathway (**Figure 4A**)—pathways critical for Treg differentiation. Analysis of TCR signaling-related genes identified *Nr4a1* as a key DEG significantly upregulated in pAkk-treated mice (**Figure 4B**), a nuclear hormone receptor known to mediate TCR-dependent Treg differentiation (28).

We validated the RNA-seq results with qRT-PCR: *in vitro* stimulation of naïve CD4^+^ T cells with pAkk (MOI = 100) significantly upregulated *Nr4a1* expression (≈2-fold) compared to the control group, while live Akk had no effect (**Figure 4C**). *In vivo*, pAkk administration significantly increased *Nr4a1* mRNA expression in the colon tissues of HFD-fed mice compared to the control and live Akk groups (**Figure 4D**). Immunofluorescence co-staining of colon tissues confirmed increased Nr4a1^+^Foxp3^+^ double-positive cells in pAkk-treated mice (**Figures 4E, F**) indicating colocalization of Nr4a1 and Foxp3 in intestinal Tregs.

To determine the functional role of Nr4a1 in pAkk-induced Treg differentiation, we used Nr4a1^^-^/^-^^ mice (genotyping in **Supplementary Figures 4A, B**). Flow cytometry analysis showed that pAkk administration failed to increase the frequency of CD4^+^CD25^+^Foxp3^+^ Tregs in the cLP of Nr4a1^^-^/^-^^ mice, while it significantly induced Treg differentiation in WT littermates (**Figures 4G, H**). Immunofluorescence staining confirmed the absence of pAkk-induced Foxp3^+^ Treg infiltration in the colon tissues of Nr4a1^^-^/^-^^ mice (**Figures 4I, J**). Coculture experiments showed that Tregs from pAkk-treated Nr4a1^^-^/^-^^ mice lost the ability to inhibit osteoclast differentiation, while Tregs from pAkk-treated WT mice retained strong osteoclast-suppressive activity (**Figures 4K, L**). These results demonstrate that Nr4a1 is essential for pAkk-mediated intestinal Treg differentiation and subsequent osteoclast inhibition.

### 3.5 Nr4a1 knockout blocks the bone-protective effects of pAkk in HFD-induced obese mice

**Figure 5:**
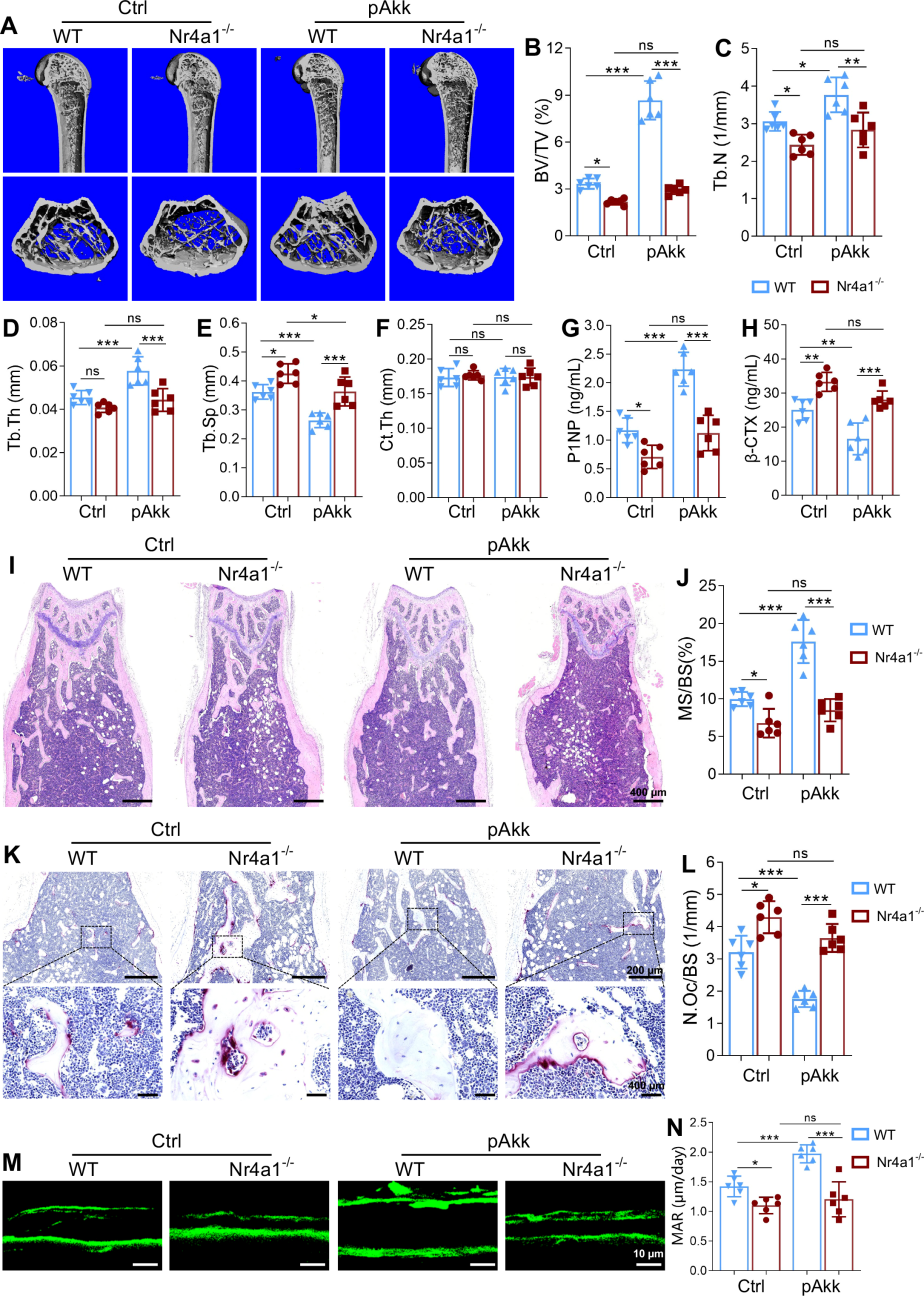
Deletion of Nr4a1 blocked the inhibition of osteoclastogenesis induced by pAkk. Nr4a1KO (Nr4a1^-/-^) and wild-type (WT) mice were fed with high-fat diet (HFD) for 12 weeks, and further administered with pAkk by daily oral gavage for 4 weeks. **(A)** Representative images of femoral bone structures assessed by μCT. **(B–F)** μCT measurements of trabecular bone volume fraction (BV/TV), trabecular number (Tb.N), trabecular thickness (Tb.Th), trabecular separation (Tb.Sp), and cortical thickness (Ct.Th). **(G, H)** Serum levels of N-terminal propeptide of type I procollagen (P1NP), and β-terminal telopeptide (β-CTX) measured by ELISA. **(I)** Representative images of hematoxylin and eosin (H&E) staining of the distal femur bone in HFD mice administered with Akk, pAkk, and the vehicle control (Scale bar: 400 μm). **(J)** Histomorphometric indices of mineralizing surface/bone surface (MS/BS). **(K)** Representative images of tartrate resistant acid phosphatase (TRAP)-stained sections of the distal femur bone, and a higher magnification was shown in lower panel (Scale bar: upper panel 100 μm, lower panel 40 μm). **(L)** Histomorphometric indices of osteoclasts per bone surface (N.Oc/BS). **(M)** Trabecular calcein double-fluorescence labeling images (green) of the representative sections (Scale bar: 10 μm). **(N)** Quantification of mineral apposition rate (MAR). Each dot indicates an individual mouse (n = 6 per group). Data are shown as means ± SEM and analyzed by one-way ANOVA followed by Bonferroni *post hoc* test. ns, not significant; *P < 0.05; **P < 0.01, and ***P < 0.001.

Finally, we verified the *in vivo* role of Nr4a1 in pAkk-mediated bone protection in HFD-fed mice.μCT analysis revealed that Nr4a1^^-^/^-^^ mice exhibited significant trabecular bone loss compared to WT littermates (regardless of pAkk treatment), with reduced BV/TV and Tb.N, and increased Tb.Sp (**Figures 5A–E**); Ct.Th was unaltered between Nr4a1^^-^/^-^^ and WT mice (**Figure 5F**). Notably, pAkk administration failed to improve bone mass or trabecular microarchitecture in Nr4a1^^-^/^-^^ mice, whereas it exerted a significant bone-protective effect in WT mice (**Figures 5A–E**). Serum ELISA assays showed that pAkk had no effect on P1NP and β-CTX levels in Nr4a1^^-^/^-^^ mice, in contrast to its ability to increase P1NP and decrease β-CTX in WT mice (**Figures 5G, H**).

Histological analysis confirmed the loss of pAkk-mediated bone protection in Nr4a1^^-^/^-^^ mice: H&E staining revealed disrupted trabecular bone structures in Nr4a1^^-^/^-^^ mice, and pAkk treatment had no restorative effect (**Figure 5I**), with no significant difference in MS/BS between pAkk-treated and control Nr4a1^^-^/^-^^ mice (**Figure 5J**). TRAP staining showed more multinucleated osteoclasts in the distal femur of Nr4a1^^-^/^-^^ mice compared to WT mice, and pAkk treatment failed to reduce osteoclast number (N.Oc/BS) in Nr4a1^^-^/^-^^ mice (**Figures 5K, L**). Dynamic bone histomorphometry showed that pAkk-induced increases in MAR were completely abrogated in Nr4a1^^-^/^-^^ mice (**Figures 5M, N**). Collectively, these results confirm that Nr4a1 is a critical mediator of the bone-protective effects of pAkk in HFD-induced obese mice.

## 4 Discussion

In this study, we demonstrate that obesity-related gut microbiota contributes to bone loss in mice and is accompanied by a reduction in intestinal *Akkermansia muciniphila* abundance. We further identify pasteurized *Akkermansia muciniphila* (pAkk), rather than live Akk, as an effective postbiotic intervention that alleviates HFD-induced trabecular bone loss. Mechanistically, pAkk promoted CD4^+^CD25^+^Foxp3^+^ Treg differentiation in the intestinal lamina propria and peripheral immune compartments, and these Tregs suppressed osteoclastogenesis. Transcriptomic and functional analyses further showed that this effect was dependent on the nuclear hormone receptor Nr4a1, as genetic deletion of Nr4a1 abolished both Treg induction and the bone-protective effects of pAkk. Together, these findings support a gut microbiota–intestinal immunity–bone remodeling axis in which pAkk likely engages intestinal CD103^+^ dendritic cells, enhances Nr4a1-dependent Treg differentiation, and subsequently inhibits osteoclast activity.

Heat-killed probiotics or postbiotics have attracted increasing attention as therapeutic candidates for metabolic and skeletal disorders because of their greater stability, safety, and translational feasibility compared with live bacteria (29, 30). In the present study, pAkk showed a clear advantage over live Akk in rescuing obesity-related bone impairment. This difference may reflect altered exposure or preservation of immunologically active bacterial components after pasteurization, leading to enhanced host recognition and downstream immune regulation (31). Our findings are broadly consistent with the concept that microbiota-targeted interventions can influence skeletal homeostasis through osteoimmune mechanisms. At the same time, previous reports on pAkk and bone are not fully concordant. A pilot clinical study reported reduced Akk abundance in patients with osteopenia, although the difference was not statistically significant (32), and preclinical studies have shown that Akk-derived extracellular vesicles attenuate ovariectomy-induced osteoporosis (33) and that Akk/Amuc_1100 reduces periodontal alveolar bone loss (11, 34). In particular, Lawenius et al. reported that pAkk did not protect against ovariectomy-induced bone loss and even reduced bone mass in gonadal intact female mice (12). This discrepancy likely reflects important differences in disease context, sex, and pathophysiology, since the present study examined male HFD-induced obesity, a model characterized by chronic metabolic inflammation, gut dysbiosis, and immune imbalance (35, 36), whereas ovariectomy models primarily reflect acute estrogen deficiency. This context-dependent distinction should be considered when interpreting the skeletal effects of pAkk across studies.

A central finding of this study is that pAkk promotes intestinal and systemic Treg differentiation and thereby suppresses osteoclastogenesis. This is biologically plausible because the gastrointestinal tract is a major immune organ, and commensal microbes are known to shape peripheral tolerance and Treg development (37). Our data extend prior work on Akk-mediated immune regulation by showing that, in obesity-associated bone loss, pAkk enhances Treg differentiation through a mechanism linked to intestinal CD103^+^ dendritic cells and the Nr4a1–Foxp3 axis. In addition to the marked reduction in osteoclast activity, pAkk treatment also increased bone formation-related indices, including P1NP, MS/BS, and MAR. Although our mechanistic data primarily support inhibition of osteoclastogenesis as the dominant pathway, these findings raise the possibility that pAkk-induced immune changes may also help create a microenvironment favorable for bone formation. Treg-associated cytokines such as IL-10 and TGF-β may contribute not only to anti-resorptive effects but also potentially to osteoblast-supportive or anabolic responses. In particular, IL-10 has been reported to support osteogenesis mainly through anti-inflammatory and immunomodulatory effects, whereas TGF-β can regulate osteoblast-lineage differentiation in a context-, dose-, and stage-dependent manner (38–40). Because direct osteoblast-targeted experiments were beyond the scope of the present study, this should be considered a plausible interpretation rather than a demonstrated mechanism.

Beyond the gut–immune–bone axis examined here, pAkk may also exert broader metabolic and anti-inflammatory actions in other obesity-affected tissues. Previous studies have shown that *Akkermansia muciniphila*, particularly in pasteurized form or via its derived components, can improve gut barrier function, reduce metabolic endotoxemia, alleviate white adipose tissue inflammation, and attenuate hepatic steatosis and liver injury in obesity-related models (41–43). These extra-skeletal effects suggest that the bone-protective activity observed in our study may occur within a wider context of systemic metabolic improvement and inflammatory attenuation. However, the present work was specifically designed to define the intestinal immune mechanism underlying bone protection, and we therefore did not directly evaluate adipose tissue inflammation, hepatic responses, or other peripheral compartments. Future studies should determine whether tissue-specific anti-inflammatory effects outside the intestine also contribute to the skeletal benefits of pAkk.

In addition to modulating host immunity, pAkk may also influence the intestinal microbial ecosystem itself. *Akkermansia muciniphila* is a mucus-associated symbiont closely linked to mucosal barrier maintenance and host–microbiota homeostasis (44), and prior studies have suggested that Akk- or pAkk-related interventions reshapes the structure of intestinal microbiota, alleviates high-fat diet-induced microbial dysbiosis, maintains intestinal microecological balance, and further enhances the stability of intestinal immune regulation (45, 46). In the present study, however, our microbiota analysis was limited to the comparison between ND- and HFD-fed mice, which established that obesity-related dysbiosis was associated with reduced Akk abundance. We did not perform microbiota profiling after pAkk administration. Therefore, although pAkk may help reshape the intestinal microenvironment, our current data do not allow us to determine whether remodeling of the overall gut microbial community contributes to the observed bone-protective phenotype. This remains an important question for future investigation.

Mechanistically, RNA-seq identified Nr4a1 as a key gene induced by pAkk, and both *in vitro* and *in vivo* experiments supported its role in Treg differentiation. Nr4a1 is a ligand-independent nuclear receptor that has been implicated in TCR-responsive immune regulation, making it a plausible molecular node linking microbial stimulation to Foxp3 induction (47, 48). However, the upstream signal responsible for activating the Nr4a1/Treg axis remains undefined. Although we observed increased colocalization between CD103^+^ dendritic cells and Foxp3^+^ cells and found that pAkk was functionally associated with CD103^+^ DC-mediated Treg induction, the precise proximal triggers—such as specific surface proteins, metabolites, cell wall-associated components, Amuc_1100, or pattern-recognition receptor pathways—were not resolved in this study. It is therefore more likely that pAkk acts upstream by modulating antigen presentation, T-cell receptor signaling, and/or innate immune sensing rather than by directly binding Nr4a1. Identification of the responsible receptor–ligand pathway will be an important direction for future work.

This study has several limitations. First, we only investigated male mice, which limits the generalizability of our findings given the known sexual dimorphism in bone metabolism and obesity-associated skeletal responses. Future studies should validate these findings in female mice and, ultimately, in human cohorts. Second, although our data strongly support a Nr4a1-dependent Treg mechanism, the precise upstream molecular trigger of pAkk remains unresolved. Third, we focused primarily on intestinal immune regulation and did not systematically evaluate other obesity-affected tissues or the post-treatment gut microbiota. Fourth, although the increase in bone formation parameters suggests a broader remodeling benefit, the direct effects of pAkk-induced immune signals on osteoblast lineage cells were not examined. Addressing these questions will be important for defining the full therapeutic potential of pAkk in obesity-related skeletal disease.

## 5 Conclusion

In conclusion, our study demonstrates that pasteurized *Akkermansia muciniphila* alleviates HFD-induced bone loss in mice via a Nr4a1-dependent mechanism involving the induction of intestinal Treg differentiation and subsequent osteoclast inhibition. These findings identify pAkk as a promising postbiotic for the treatment of obesity-related bone loss and reveal Nr4a1 as a critical molecular target for linking gut microbiota to bone homeostasis. Our work provides a novel rationale for developing microbiota-targeted therapies for skeletal disorders associated with obesity and metabolic dysfunction.

## Data Availability

The raw sequencing data of 16S rRNA gene sequencing and RNA sequencing have been deposited in the NCBI Sequence Read Archive (SRA) database under the accession numbers PRJNA1031414 (16S rRNA) and PRJNA1049627 (RNA-seq). All other data supporting the findings of this study are available from the corresponding authors upon reasonable request.

## Glossary

Akk: *Akkermansia muciniphila*
ANOVA: analysis of variance
ARRIVE: Animal Research, Reporting *In Vivo* Experiments
BMMs: bone marrow monocytes
BSA: bovine serum albumin
BV/TV: bone volume fraction
β-CTX: β-isomer of C-terminal telopeptide of type I collagen
cLP: colonic lamina propria
CFU: colony-forming unit
Ct.Th: cortical thickness
DCs: dendritic cells
DEGs: differentially expressed genes
DAPI: 4′,6-diamidino-2-phenylindole
EDTA: ethylenediaminetetraacetic acid
ELISA: enzyme-linked immunosorbent assay
FACS: fluorescence-activated cell sorting
FDR: false discovery rate
FMT: fecal microbiota transplantation
GO: Gene Ontology
GMT: gut microbiota transplantation
GMT^ND^: gut microbiota transplantation from normal diet-fed donors
GMT^HFD^: gut microbiota transplantation from high-fat diet-fed donors
H&E: hematoxylin and eosin
HFD: high-fat diet
KEGG: Kyoto Encyclopedia of Genes and Genomes
MAR: mineral apposition rate
M-CSF: macrophage colony-stimulating factor
MMA: methyl methacrylate
MOI: multiplicity of infection
MS/BS: mineralizing surface per bone surface
μCT: micro-computed tomography
N.Oc/BS: osteoclast number per bone surface
ND: normal diet
Nr4a1: nuclear receptor subfamily 4 group A member 1
OCT: optimal cutting temperature
OTUs: operational taxonomic units
PBS: phosphate-buffered saline
P1NP: procollagen type I N-terminal propeptide
pAkk: pasteurized *Akkermansia muciniphila*
PCR: polymerase chain reaction
PLP: periodate-lysine-paraformaldehyde
qRT-PCR: quantitative real-time polymerase chain reaction
RANKL: receptor activator of nuclear factor-κB ligand
RBC: red blood cell
RNA-seq: RNA sequencing
ROI: region of interest
SEM: standard error of the mean
SMI: structure model index
SPF: specific pathogen-free
Tb.N: trabecular number
Tb.Sp: trabecular separation
Tb.Th: trabecular thickness
TCR: T-cell receptor
Tgfb1: transforming growth factor beta 1
Tgfb2: transforming growth factor beta 2
TRAP: tartrate-resistant acid phosphatase
Tregs: regulatory T cells
WT: wild type.
