# Supplementary material for: Pasteurized Akkermansia muciniphila alleviates high-fat diet-induced bone loss via Nr4a1-dependent Treg differentiation
Source: Front Immunol. 2026 May 22;17:1833607. doi: 10.3389/fimmu.2026.1833607 (PMC13236502; doi:10.3389/fimmu.2026.1833607)
Supplement: Supplementary file 1 [file DataSheet1.pdf]

Supplementary Material

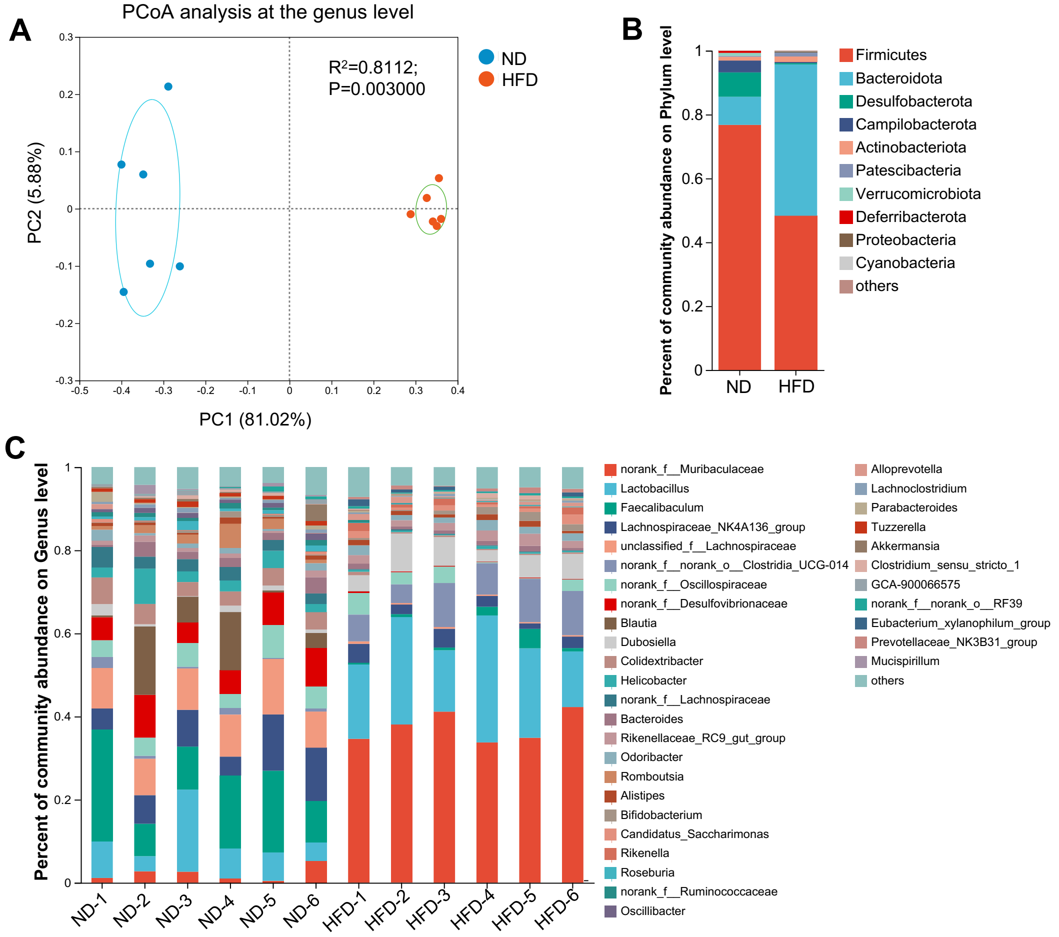

Supplementary Figure 1. HFD changed the gut microbiota composition. (A) Principal co-ordinates analysis (PCoA) of fecal microbiota from mice in the ND and HFD groups using Bray-Curtis distances (ANOSIM test, n = 6 per group). (B) Relative abundance of bacterial groups on the phylum level in the ND and HFD groups (n = 6 per group). (C) Relative abundance of bacterial groups on the genus level in the ND and HFD groups (top35 genus were exhibited, n = 6 per group).

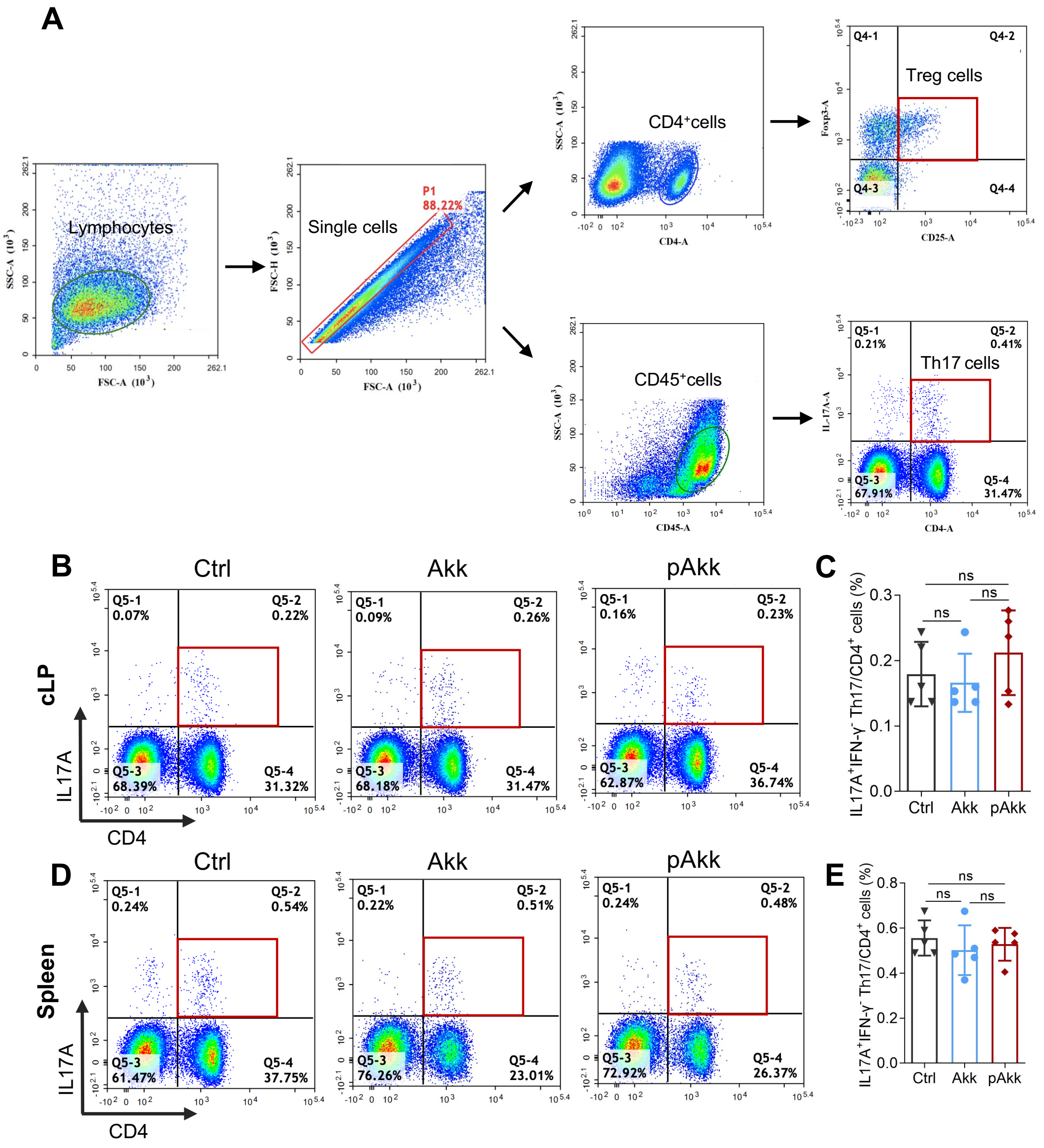

Supplementary Figure 2. Supplementation with pasteurized Akk had no effects on Th17 differentiation. (A) Gating strategy for flow cytometric analyses. Lymphocytes were gated based on size and granularity and then doublets were excluded. Treg cells were selected using CD4, CD25and Foxp3 markers, Th17 cells were selected using CD45, CD4 and IL17 markers. (B, C) Representative plots and percentage of CD4$^+$IL17$^+$ Th17 cells of colonic lamina propria (cLP) from HFD mice administrated with Akk, pasteurized Akk (pAkk), and the control assessed using flowcytometry. (D, E) Representative plots and percentage of CD4$^+$IL17$^+$ Th17 cells of spleen from HFD mice administrated with Akk, pAkk, and the control assessed using flowcytometry. Data are shown as shown as means ± SEM, and analyzed by one-way ANOVA followed by Bonferroni post hoc test. Each dot indicates an individual mouse (n = 5 per group) and $P < 0.05$ was considered as statistically significant.

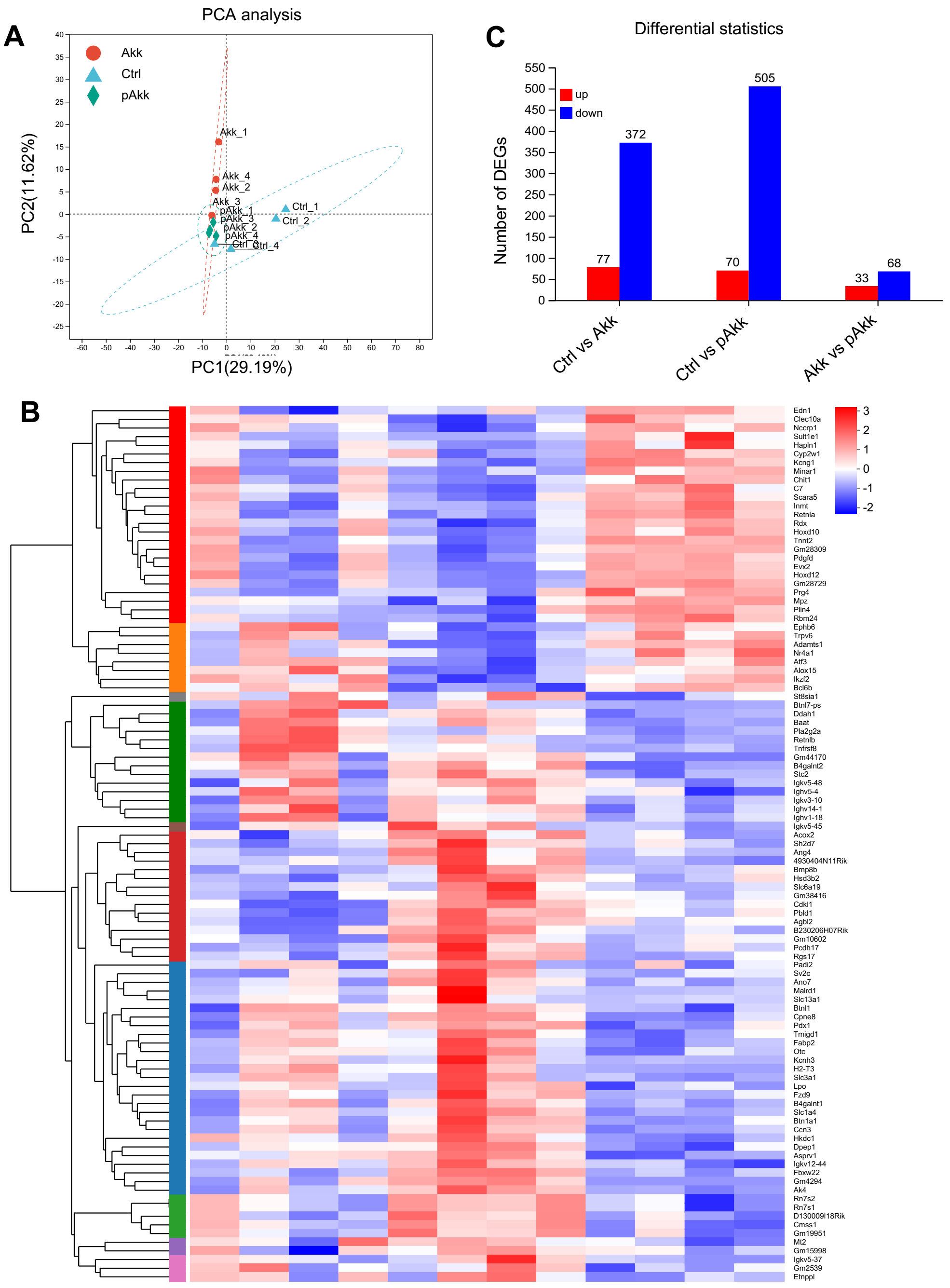

Supplementary Figure 3. Supplementation with pasteurized Akk altered the gene expression of colonic tissues. (A) Principal component analysis (PCA) shows clustering of colonic samples from control, Akk and pAkk groups. PC1, explaining 11.62% of the total variance, PC2, explaining 29.19% of the total variance. (B) Cluster heatmap of differentially expressed genes (DEGs) among H control, Akk and pAkk groups. Rows represent the differential genes, columns represent different groups, and boxes in blue or red represent lower and higher expression level, respectively. (C) Bar plots of the number of differentially expressed genes (DEGs) identified in pairwise comparison among control, Akk and pAkk groups.

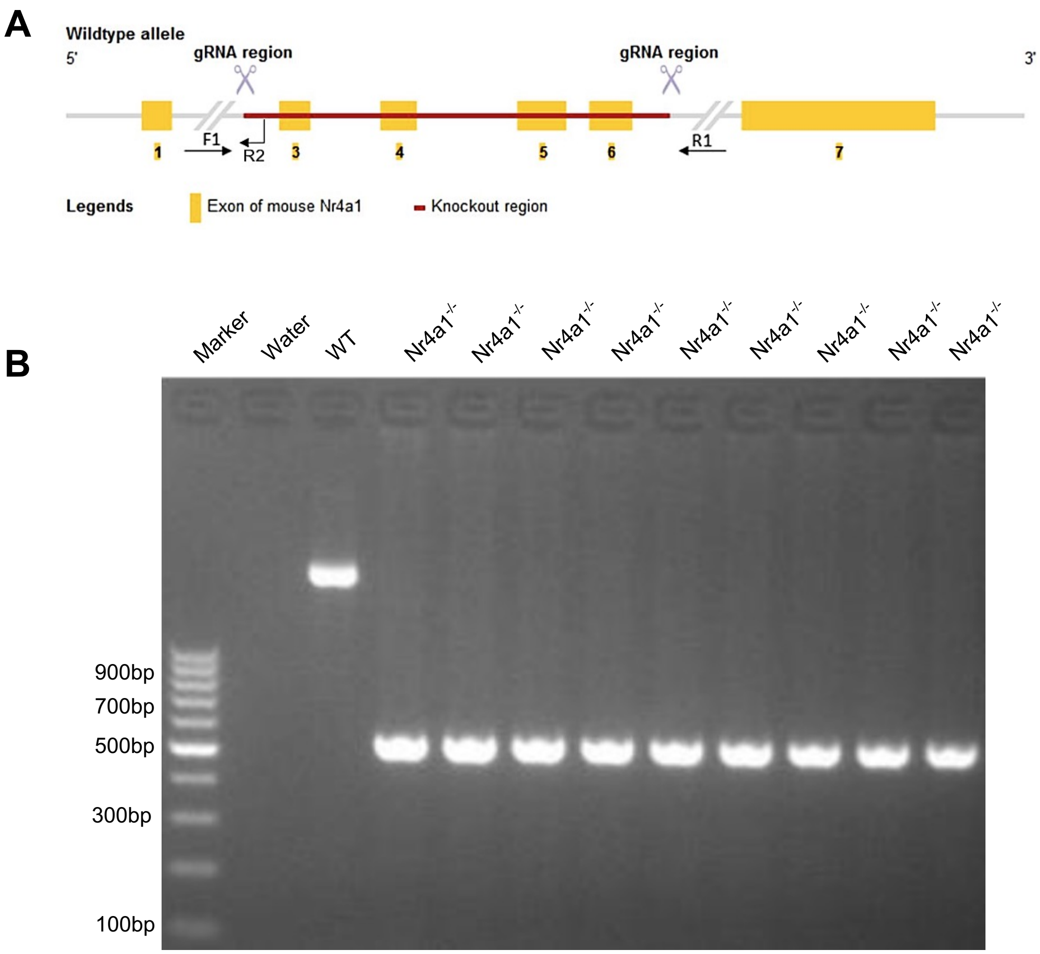

Supplementary Figure 4. Identification of Nr4a1 knockout mice. (A) Strategy of genotyping. Exon of mouse *Nr4a1* was knockout using CRISPR-Cas9 system. (B) DNA agarose gel electrophoresis of genomic DNA from mouse tail. Wild-type (WT) mice showed a band at 2,577 bp, and Nr4a1 KO homozygous mice (Nr4a1$^{-/-}$) mice showed a band at 532 bp.

Table S1 Primer sequences for real- time PCR

Gene	Primer Sequencing（5‘-3’）
IL-10	F：GCTCTTACTGACTGGCATGAG
	R：CGCAGCTCTAGGAGCATGTG
Tgfb1	F：CTCCCGTGGCTTCTAGTGC
	R：GCCTTAGTTTGGACAGGATCTG
Tgfb2	F：TCGACATGGATCAGTTTATGCG
	R：CCCTGGTACTGTTGTAGATGGA
Nr4a1	F：CGGACAGACAGCCTAAAAGG
	R：TAACGTCCAGGGAACCAGAG
